# How economic weights translate into genetic and phenotypic progress, and vice versa

**DOI:** 10.1186/s12711-023-00807-0

**Published:** 2023-06-08

**Authors:** Henner Simianer, Johannes Heise, Stefan Rensing, Torsten Pook, Johannes Geibel, Christian Reimer

**Affiliations:** 1 Animal Breeding Group, Department of Animal Sciences, Center for Integrated Breeding Research, University of Goettingen, Albrecht-Thaer-Weg 3, 37075 Göttingen, Germany; 2 IT Solutions for Animal Production (vit), Heinrich-Schröder-Weg 1, 27283 Verden/Aller, Germany; 3 Animal Breeding and Genomics Group, Wageningen University & Research, PO Box 338, 6700 AH Wageningen, The Netherlands; 4 Friedrich-Loeffler-Institut, Division of Farm Animal Genetics, Höltystraße 10, 31535 Neustadt, Germany

## Abstract

**Background:**

This paper highlights the relationships between economic weights, genetic progress, and phenotypic progress in genomic breeding programs that aim at generating genetic progress in complex, i.e., multi-trait, breeding objectives via a combination of estimated breeding values for different trait complexes.

**Results:**

Based on classical selection index theory in combination with quantitative genetic models, we provide a methodological framework for calculating expected genetic and phenotypic progress for all components of a complex breeding objective. We further provide an approach to study the sensitivity of the system to modifications, e.g. to changes in the economic weights. We propose a novel approach to derive the covariance structure of the stochastic errors of estimated breeding values from the observed correlations of estimated breeding values. We define ‘realized economic weights’ as those weights that would coincide with the observed composition of the genetic trend and show, how they can be calculated. The suggested methodology is illustrated with an index that aims at achieving a breeding goal composed of six trait complexes, that was applied in German Holstein cattle breeding until 2021.

**Conclusions:**

Based on the presented results, the main conclusions are (i) the composition of the observed genetic progress matches the expectations well, with predictions being slightly better when the covariance of estimation errors is taken into account; (ii) the composition of the expected phenotypic trend deviates significantly from the expected genetic trend due to the differences in trait heritabilities; and (iii) the realized economic weights derived from the observed genetic trend deviate substantially from the predefined ones, in one case even with a reversed sign. Further results highlight the implications of the change to a modified breeding goal based on the example of a new index comprising eight, partly new, trait complexes, which is used since 2021 in the German Holstein breeding program. The proposed framework and the analytical tools and software provided will be useful to define more rational and generally accepted breeding objectives in the future.

## Background

Modern livestock breeding programs aim at improving several traits at the same time. Typically, the considered traits have a polygenic background and, with few exceptions (see e.g. [1]), the aim is to shift the population in a certain direction, rather than keeping a trait in an optimal range.

Following [2], the definition of breeding goals addresses the question ‘where to go’, while breeding programs describe ‘how to get there’. According to the conceptual definition of breeding programs suggested by Simianer et al. [3], defining the breeding goal is not considered as an intrinsic part of a breeding program, but as an external input. The natural framework for the proportional allocation of genetic progress among a set of breeding objectives is incorporated in the theory of selection indices [4], in which weights to the different traits in the breeding goals are allocated on an economic basis [5]. Determination of those economic weights is challenging and can be achieved with various methodological approaches, as reviewed by [6]. However, how does the composition of economic weights affect the composition of the genetic and phenotypic trend? While in a simple setting (e.g. when selection is based on the selection candidates’ own performance) the derivation of the composition of the expected genetic progress with a given set of economic weights is rather straightforward, things become slightly more complicated in real life settings, as illustrated, e.g. for a pig breeding scenario by [7].

In this contribution, we will consider the case of genomic selection in dairy cattle breeding, where genetic progress is mainly driven by the selection of young bulls based on their genomic breeding values. All candidate bulls have genomic breeding values for the same set of traits and with very similar reliability, because most of the information stems from genomic sources which is uniform among genotyped individuals. The reliability of genomic breeding values is primarily a function of population parameters and technical parameters of the genomic breeding value estimation scheme, like training set size and marker density [8], and depends only to a small extent on the characteristics of the actual individual. Minor differences in reliabilities of individual breeding values may result from differences in the amount of ancestral information in a two-step or single-step [9] approach. In most dairy cattle breeding programs, breeding values of different traits are combined into an overall index, which reflects the breeding goal in the respective breeding program, and it is assumed that this overall index is the main criterion used for selection [10]. Often, other traits exist, which are not included in the index, but for which the correlated selection response might still be of interest.

The main questions, which we want to address here are:

- Given a certain index, and assuming that genetic progress is primarily due to the selection of young bulls based on this index, which composition of genetic and phenotypic progress do we expect (both for the traits in the index and additional traits)?
- Given an observed pattern of genetic progress for a set of traits, what are the ‘realized’ economic weights that would coincide with the observed composition of the genetic trend?
- How sensitive is the composition of the genetic trend to variations in the composition of the index?
- What are the consequences of a change in the breeding goal?

We will study this problem with a classical selection index approach, in which we assume that each individual has two sets of traits: for $n$ different traits, it has a true breeding value, and for a subset of $m$ of these traits, it also has an estimated breeding value. We call those $m$ traits ‘index traits’ hereafter. The main focus will be the specification of the appropriate variance and covariance matrices for the described setting based on classical quantitative genetics theory [11]. With those, we construct a selection index for which we assume that selection will be on a combined index of the estimated breeding values, and we will derive the selection response in the set of true breeding values, which is the genetic trend, and the corresponding phenotypic trend. We further present an approach to study the sensitivity of the index to changes in the composition of economic weights. With this and the provision of a corresponding R package, our aim is to expand the toolkit for applied animal breeders, in particular to help them assess the practical consequences of a change in breeding objective.

## Methods

### Specification of the model

We consider the case where we have $n$ traits that are part of the breeding goal. These traits, which we call ‘breeding goal traits’, are assigned an economic value in the definition of the total merit, which is the true, but unobservable, genetic value of an individual. We further assume that for all these traits, or a subset comprising $m \le n$ of these traits, estimated breeding values are available, which conceptually are considered as observable traits with a reliability as estimated by the genetic evaluation system. These quasi-phenotypes can be combined appropriately to provide an estimate of the unobservable total merit via classical selection index theory. These traits are thus called ‘index traits’.

For the $n$ breeding goal traits, the unobservable true breeding values of an individual are distributed as $\mathbf{u}\sim (0,{\varvec{\Gamma}})$, where ${\varvec{\Gamma}}$ is the $n\times n$ additive genetic variance–covariance matrix.

To provide a general approach to select $m$ index traits out of a total of the $n$ breeding goal traits represented in vector $\mathbf{u}$, we define an $m \times n$ matrix $\mathbf{D}$ with element ${d}_{ij}$ being 1 if trait $j$ in the set of breeding goal traits is identical to index trait $i$, and 0 otherwise. With this, the vector of true breeding values for the index traits is $\mathbf{v}=\mathbf{D}\mathbf{u}$ and the genetic variance–covariance matrix of the index traits can be constructed from the variance–covariance matrix of all traits as $VCV\left(\mathbf{v}\right)=\mathbf{D}{\varvec{\Gamma}}{\mathbf{D}}^{\mathbf{^{\prime}}}.$ Note that if the set of index traits is identical to the set of breeding goal traits ($n=m$), $\mathbf{D}$ is the identity matrix.

Estimated breeding values have a reliability lower than 1, which must be taken into account [12]. To do this, we model the vector of estimated breeding values for the index traits in two steps: first, we introduce an ‘unscaled’ proxy of the estimated breeding value, which already has the correct correlation with the true breeding value, and in the second step, we rescale this unscaled breeding value so that it has the correct distributional properties. We model the vector of ‘unscaled’ estimated breeding values $\mathbf{q}$ as sum of the true breeding value plus an estimation error, i.e., $\mathbf{q}=\mathbf{v}+\mathbf{e}$, where $\mathbf{e}$ is a vector of $m$ residual errors with $\mathbf{e}\sim (\boldsymbol{0},\mathbf{E})$ where $\mathbf{E}$, for the time being, is assumed to be a diagonal matrix so that the errors for different traits are uncorrelated. We further assume that, in general, the errors are independent of the true breeding values (i.e., $Cov(\mathbf{v},\mathbf{e})=\boldsymbol{0}$), for a detailed discussion of this assumption see [13].

The reliability of a breeding value is defined as the squared correlation between true and estimated breeding values, i.e., ${\rho }_{{v}_{i},{q}_{i}}^{2}=\frac{{\gamma }_{ii}^{2}}{{\gamma }_{ii}\times ({\gamma }_{ii}+{\varepsilon }_{ii})}=\frac{{\gamma }_{ii}}{{\gamma }_{ii}+{\varepsilon }_{ii}}$, where ${\gamma }_{ii}$ and ${\varepsilon }_{ii}$ are the diagonal elements of $\mathbf{D}{\varvec{\Gamma}}\mathbf{D}\mathbf{^{\prime}}$ and $\mathbf{E}$ pertaining to index trait $i$, respectively. From this follows that ${\varepsilon }_{ii}=\frac{1-{\rho }_{{v}_{i},{q}_{i}}^{2}}{{\rho }_{{v}_{i},{q}_{i}}^{2}}{\gamma }_{ii}$, assuming the reliability and the true genetic variance for trait $i$ are known. Consequently, the variance of the unscaled estimated breeding value is:

$$Var\left({q}_{i}\right)=Var\left({v}_{i}\right)+Var\left({e}_{i}\right)={\gamma }_{ii}+\frac{1-{\rho }_{{v}_{i},{q}_{i}}^{2}}{{\rho }_{{v}_{i},{q}_{i}}^{2}}{\gamma }_{ii}=\frac{1}{{\rho }_{{v}_{i},{q}_{i}}^{2}}{\gamma }_{ii}.$$

Note that with this, $Var\left({q}_{i}\right)> Var\left({v}_{i}\right)$, while quantitative genetics theory suggests that, through regression to the mean [14], the variance of estimated breeding values must be smaller than the variance of the true breeding values, or, more precisely, the variance of the estimated breeding values for trait $i$ must be equal to the product of the reliability of the estimated breeding value $i$ times $Var\left({v}_{i}\right)$.

This can be achieved by re-scaling the estimated breeding values in the form $\mathbf{c}=\mathbf{R}\mathbf{q}$, where $\mathbf{R}$ is an $m\times m$ diagonal matrix with the respective reliabilities on the diagonal, i.e., diagonal element $i$,$i$ of matrix $\mathbf{R}$ being ${r}_{ii}={\rho }_{{v}_{i},{q}_{i}}^{2}$.

Then, $Var\left(\mathbf{c}\right)=\mathbf{C}=\mathbf{R}\bigg (\mathbf{D}{\varvec{\Gamma}}\mathbf{D}\mathrm{^{\prime}}+\mathbf{E}\bigg)\mathbf{R}$ with diagonal elements ${c}_{ii}=({{\rho }_{{v}_{i},{q}_{i}}^{2})}^{2}\times \frac{1}{{\rho }_{{v}_{i},{q}_{i}}^{2}}{\gamma }_{ii}={\rho }_{{v}_{i},{q}_{i}}^{2}{\gamma }_{ii}$. Note that all phenotypic correlations under this model are exclusively due to genetic covariances between traits.

With this, the total genetic merit can be defined as $T={\mathbf{w}}^{\mathbf{^{\prime}}}\times \mathbf{u}$, where $\mathbf{w}$ is a vector of length $n$ comprising relative economic weights for the breeding goal traits. An estimate of the total genetic merit can be obtained with the index $I={\mathbf{b}}^{\mathbf{^{\prime}}}\times \mathbf{c}$, where $\mathbf{b}$ is a vector of length $m$ comprising the unknown index weights for all index traits. Selection index theory [4, 15] suggests that the optimal index weights, such that the correlation between $T$ and $I$ (${\rho }_{TI}$) is maximized, are obtained from the selection index normal equations:

$$Var\left(\mathbf{c}\right)\times \mathbf{b}=Cov\left(\mathbf{c},\mathbf{u}\right)\times \mathbf{w}.$$

With $Cov\left(\mathbf{c},\mathbf{u}\right)=Cov\left(\mathbf{R}\left(\mathbf{D}\mathbf{u}+\mathbf{e}\right),\mathbf{u}\right)=\mathbf{R}\mathbf{D}{\varvec{\Gamma}}$, we obtain the selection index normal equations:

$$\mathbf{R}\left(\mathbf{D}{\varvec{\Gamma}}\mathbf{D}\mathbf{^{\prime}}+\mathbf{E}\right)\mathbf{R}\times \mathbf{b}=\mathbf{R}\mathbf{D}{\varvec{\Gamma}}\times \mathbf{w},$$

from which the optimum index weights can be calculated as:

1 $$\mathbf{b}={\bigg(\mathbf{R}\left(\mathbf{D}{\varvec{\Gamma}}\mathbf{D}\mathbf{^{\prime}}+\mathbf{E}\right)\mathbf{R}\bigg)}^{-\boldsymbol{1}}\mathbf{R}\mathbf{D}{\varvec{\Gamma}}\times \mathbf{w}.$$

Note that the matrices resulting from this derivation are identical to those suggested by Miesenberger [16], which, however, is a PhD-thesis in German language, published to our knowledge solely on paper and thus is not generally accessible.

### Estimation of the variance–covariance matrix of residuals

Remember that it was assumed that the residuals of the estimated genomic breeding values are uncorrelated, i.e., that $\mathbf{E}$ is a diagonal matrix. It has been previously argued [7, 13, 17] that this is not necessarily the case, and we provide in the following an approach to derive this covariance structure from empirical data and how to integrate it into the model.

Let us assume that we have an empirical set of estimated breeding values $\widehat{\mathbf{c}}$ for the *m* index traits for an unselected sample of contemporary breeding animals. The variance–covariance-matrix of these breeding values $Var\left(\widehat{\mathbf{c}}\right)=\mathbf{H}$ can be empirically determined. Under the model defined above, $E\left(\mathbf{H}\right)=\mathbf{R}\left(\mathbf{D}{\varvec{\Gamma}}{\mathbf{D}}^{\mathbf{^{\prime}}}+{\varvec{\Sigma}}\right)\mathbf{R}$, where ${\varvec{\Sigma}}$ is the variance–covariance matrix of the residuals of the estimated genomic breeding values without the restriction that these are uncorrelated. From this, we can obtain an estimate of the residual variance–covariance matrix ${\varvec{\Sigma}}$ by de-regressing the empirical variance–covariance matrix $\mathbf{H}$ and subtracting the genetic variance–covariance matrix, which is assumed to be known:

2 $$\widehat{{\varvec{\Sigma}}}={\mathbf{R}}^{-\boldsymbol{1}}\mathbf{H}{\mathbf{R}}^{-\boldsymbol{1}}-\mathbf{D}{\varvec{\Gamma}}{\mathbf{D}}^{\mathbf{^{\prime}}}.$$

The matrix $\mathbf{E}$ now can be replaced by $\widehat{{\varvec{\Sigma}}}$ in Eq. (1), yielding:

3 $$\mathbf{b}={\bigg(\mathbf{R}\left(\mathbf{D}{\varvec{\Gamma}}\mathbf{D}\mathbf{^{\prime}}+\widehat{{\varvec{\Sigma}}}\right)\mathbf{R}\bigg)}^{-\boldsymbol{1}}\mathbf{R}\mathbf{D}{\varvec{\Gamma}}\times \mathbf{w}.$$

Note that due to this modification, the resulting index weights in $\mathbf{b}$ and all downstream results are no longer fully equivalent to those presented by [16].

### Expected composition of the genetic trend

Following classical selection index theory, the variance of the index is:

4 $${\sigma }_{I}^{2}=Var\left(\mathbf{b}\mathbf{^{\prime}}\mathbf{c}\right)={\mathbf{b}}^{\mathbf{^{\prime}}}\mathbf{R}\left(\mathbf{D}{\varvec{\Gamma}}{\mathbf{D}}^{\mathbf{^{\prime}}}+\widehat{{\varvec{\Sigma}}}\right)\mathbf{R}\mathbf{b},$$

and the vector of the expected genetic progress, when individuals are selected based on the index with selection intensity $i$, is:

$$\mathbf{d}=\frac{\mathrm{i}}{{\upsigma }_{\mathrm{I}}}{\varvec{\Gamma}}{\mathbf{D}}^{\mathbf{^{\prime}}}\mathbf{R} \, \mathbf{b}=\frac{\mathrm{i}}{{\upsigma }_{\mathrm{I}}}{\varvec{\Gamma}}{\mathbf{D}}^{\mathbf{^{\prime}}}\mathbf{R} \, {\left(\mathbf{R}\left(\mathbf{D}{\varvec{\Gamma}}{\mathbf{D}}^{\mathbf{^{\prime}}}+\widehat{{\varvec{\Sigma}}}\right)\mathbf{R}\right)}^{-\boldsymbol{1}}\mathbf{R}\mathbf{D}{\varvec{\Gamma}}\times \mathbf{w}.$$

Applying the general rule in matrix algebra ${(\mathbf{A}\times \mathbf{B})}^{-\boldsymbol{1}}={\mathbf{B}}^{-\boldsymbol{1}}\times {\mathbf{A}}^{-\boldsymbol{1}}$, where $\mathbf{A}$ and $\mathbf{B}$ are invertible quadratic matrices, this simplifies to:

5 $$\mathbf{d}=\frac{\mathrm{i}}{{\upsigma }_{\mathrm{I}}}{\varvec{\Gamma}}{\mathbf{D}}^{\mathbf{^{\prime}}} \, {\left(\mathbf{D}{\varvec{\Gamma}}{\mathbf{D}}^{\mathbf{^{\prime}}}+\widehat{{\varvec{\Sigma}}}\right)}^{-\boldsymbol{1}}\mathbf{D}{\varvec{\Gamma}}\times \mathbf{w}.$$

Here $\mathbf{d}$ is a vector of length $n$ containing the expected changes in the true breeding values in $\mathbf{v}$ from one round of selection. Note that the expected total genetic progress in economic terms then is $\Delta G=\mathbf{d}^\prime\mathbf{w}$.

### Expected composition of the phenotypic trend

A further aspect of practical relevance is how the expected genetic trend translates into a phenotypic trend, i.e., what changes in phenotypes can actually be expected for the different traits and what is the overall composition of the phenotypic trend. The expected genetic trend in trait $i$ is the i-th element of vector $\mathbf{d}$ obtained from Eq. (5), and since the regression of the phenotype on the breeding value is 1, we also would expect the phenotypic trend to be ${d}_{i}$. Since traits may be expressed on very different scales, a fair comparison of the composition of the expected phenotypic trend should be scaled in phenotypic standard deviations of the respective traits. Let us assume, trait $i$ has heritability ${h}_{i}^{2}$. Then, the phenotypic variance for this trait is ${\sigma }_{{p}_{i}}^{2}=\frac{{\gamma }_{ii} }{{h}_{i}^{2}}$ and the genetic progress expressed in phenotypic standard deviations in this trait is:

6 $${d}_{{p}_{i}}=\frac{{d}_{i}}{{\sigma }_{{p}_{i}}}=\frac{{d}_{i}\times {h}_{i}}{\sqrt{{\gamma }_{ii}}}.$$

### Sensitivity of genetic progress to changes in economic weights

We might also be interested in the question of how a change in the economic weight of some traits would affect the composition of the genetic trend. In principle, this could be studied by taking the first derivative of $\mathbf{d}$ with respect to $\mathbf{w}$. However, results may be misleading since the approach ignores the constraint that the weights in $\mathbf{w}$ must sum to 1, which means that increasing the weight of one trait must be accompanied by a decrease in the weight of some other traits.

Thus, we propose to calculate an approximate empirical derivation by contrasting the genetic trend obtained with a modified weight vector and the optimal genetic trend for each trait separately. For trait $i$, we calculate a modified vector ${\mathbf{w}}^{i}$ with elements ${w}_{i}^{i}= {w}_{i}+ \theta$ for trait $i$ and ${w}_{j}^{i}= {(1-\frac{\theta }{1-{w}_{i}})w}_{j} \,\mathrm{for \,all\, other\, traits }\,j\ne i$, where $\theta$ is a small constant. In this way, the weights of all other traits are reduced proportionally while all weights still sum to 1.

By using ${\mathbf{w}}^{i}$ in Eqs. (3), (4) and (5), respectively, we obtain new values for the vector of index weights, the variance of the index, and the vector of genetic changes in all traits, which we call ${\mathbf{d}}^{i}$. By multiplying this modified vector of genetic changes with the original weight vector $\mathbf{w}$, we obtain a new estimate of the overall genetic trend $\Delta {G}^{i}={\mathbf{d}}^{{\varvec{i}^{\prime}}}\mathbf{w}$. Note, that $\Delta {G}^{i}\le \Delta G$ because the selection index maximizes the genetic trend by design and therefore a deviation from the optimal index cannot lead to an increased overall genetic trend.

### Realized economic weights

In an ongoing breeding program, we can measure the empirical genetic progress in the set of relevant traits, i.e., those traits that have a non-zero economic weight in $\mathbf{w}$ (several examples for this are addressed in [18]). Again, we are more interested in the proportional composition of the realized genetic progress, rather than in the absolute values, so the observed vector ${\varvec{\updelta}}$ reflects the proportion of the overall genetic progress (scaled in genetic standard deviations) that is attributed to the different traits, such that $\sum \left|{\varvec{\updelta}}\right|=1$.

For the case of matrix $\mathbf{D}$ having rank $n$, i.e., if the number of index traits is equal to the number of breeding goal traits, we can derive the vector of realized economic weights ${\varvec{\upomega}}$, which is the set of hypothetical economic weights that would have led to the observed composition of genetic trend, if selection was strictly based on an index constructed with these realized economic weights.

Based on the equation:

$$\mathbf{d}=\frac{i}{{\sigma }_{I}}{\varvec{\Gamma}}{\mathbf{D}}^{\mathbf{^{\prime}}}\mathbf{R} \, \mathbf{b},$$

and omitting the scalar coefficient we can calculate the realized index weights ${\varvec{\upbeta}}$, which correspond to the observed composition of genetic progress in ${\varvec{\updelta}}$ as:

7 $${\varvec{\upbeta}}={({\varvec{\Gamma}}\mathbf{D}\mathbf{^{\prime}}\mathbf{R}\text{ )}}^{-\boldsymbol{1}}{\varvec{\updelta}}.$$

Putting these realized index weights in Eq. (3) we can solve for the realized economic weights ${{\varvec{\upomega}}}_{\mathbf{u}}$:

$${\varvec{\upomega}}={\left(\mathbf{R}\mathbf{D}{\varvec{\Gamma}}\right)}^{-\boldsymbol{1}}{\left(\mathbf{R}\left(\mathbf{D}{\varvec{\Gamma}}{\mathbf{D}}^{\mathbf{^{\prime}}}+\widehat{{\varvec{\Sigma}}}\right)\mathbf{R}\right)({\varvec{\Gamma}}\mathbf{D}\mathbf{^{\prime}}\mathbf{R}\text{ )}^{-\boldsymbol{1}}} {\varvec{\updelta}},$$

which simplifies to:

8 $${\varvec{\upomega}}={\left(\mathbf{D}{\varvec{\Gamma}}\right)}^{-\boldsymbol{1}}{\left(\mathbf{D}{\varvec{\Gamma}}{\mathbf{D}}^{\mathbf{^{\prime}}}+\widehat{{\varvec{\Sigma}}}\right)({\varvec{\Gamma}}\mathbf{D}\mathbf{^{\prime})}^{-\boldsymbol{1}}} {\varvec{\updelta}}.$$

Since we have dropped the scalar factor $i/{\sigma }_{I}$, the results must not be interpreted in absolute terms, but reflect the relative weights and might also be rescaled such that $\sum \left|{\varvec{\upomega}}\right|=1$. Our approach is similar to an index-in-retrospect, which was originally introduced by Dickerson et al. [19] and later used, for example, to analyze selection practices among North American dairy farmers [20] or to document the historical selection applied by Nguni breeders in South Africa [21], but the approach proposed here is conceptually more general.

Remember that a unique solution for ${\varvec{\upomega}}$ only is available if the matrix $\mathbf{D}$ has rank $n$. If $m < n$, regular inverses in Eqs. (7) and (8) do not exist, and consequently a unique solution for ${\varvec{\upomega}}$ cannot be obtained.

### Software

The described framework was implemented in the R package “IndexWizard”, which can be accessed from GitHub (https://github.com/johannesgeibel/IndexWizard) and will be submitted to CRAN soon. This allows researchers and professionals to easily explore the expected outcome of potential changes of the economic weights of an index and to *post-hoc* analyze whether the expected trend matches the observed trend.

## Results and discussion

We illustrate the concept with real data from the German Holstein Friesian breeding program. Here, breeding values are estimated for entire trait complexes (such as ‘milk’ or ‘fertility’), which in most cases are composed of several traits. Details can be found in the respective documentation [22]. The trait complexes are expressed as deviations from a moving population mean (set to 100), which is updated once per year, and for each trait complex, the genetic standard deviation is set to 12. Breeding values are scaled such that high values are favorable from the breeder’s perspective. For most trait complexes, estimated breeding values of individual traits are first combined to a sub-index, and then, an overall index is calculated from these combined breeding values for the trait complex. To give an example: first, breeding values for milk yield, protein yield and fat yield are estimated, then protein yield and fat yield are combined into a sub-index reflecting the trait complex ‘milk’, which, after appropriate scaling, yields the RZM. Finally, the RZM is combined with other trait complexes to the total merit index, which is called RZG. The overall index reflects the total genetic merit with respect to the breeding goal. Here, we consider two versions of such a total merit index: the ‘old index’, which was used until April 2021 and is comprised of six trait complexes. The ‘new index’, which was implemented from April 2021 onwards and comprises eight trait complexes [22]. Since the two sets of trait complexes overlap, we have in total 10 trait complexes, which are presented in Table 1 together with the respective index weights in the two indices and the respective parameters.

**Table 1:** Trait complex names and abbreviations, economic weights in the old and the new index, reliability of genomic breeding values (${{\varvec{\rho}}}_{{\varvec{i}}}^{2}$), heritabilities (${{\varvec{h}}}_{{\varvec{i}}}^{2}$) and genetic correlations among all traits

Trait complex names	Abbreviation	Economic weights		${\rho }_{i}^{2}$	${h}_{i}^{2}$	Genetic correlations								
		Old index	New index			RZN	RZEo	RZEn	RZR	RZKm	RZKd	RZH	RZC	RZS
Milk yield^a,b^	RZM	0.45	0.36	0.743	0.314	0.13	0.13	0.07	− .15	0.11	0.07	0.09	− .02	0.04
Functional herd life^a,b^	RZN	0.20	0.18	0.673	0.090		0.23	0.28	0.43	0.25	0.22	0.78	0.13	0.46
Conformation old^a^	RZEo	0.15	–	0.638	0.194			0.92	0.02	0.09	−.05	0.25	− .10	0.19
Conformation new^b^	RZEn	–	0.15	0.717	0.194				0.06	0.08	− .03	0.31	− .10	0.25
Fertility^a,b^	RZR	0.10	0.07	0.541	0.013					0.32	0.19	0.41	0.04	0.15
Calving traits maternal^a,b^	RZKm	0.03	0.015	0.635	0.049						0.00	0.25	0.04	0.13
Calving traits direct^b^	RZKd	–	0.015	0.604	0.033							0.23	0.05	0.10
Health^b^	RZH	–	0.18	0.720	0.061								0.10	0.57
Young stock survival^b^	RZC	–	0.03	0.499	0.014									0.02
Somatic cell score^a^	RZS	0.07	–	0.764	0.273									

^a^Trait complexes in the old index

^b^Trait complexes in the new index

Four of the trait complexes (RZM, RZN, RZE and RZKm) are present in both indices, albeit with different weights, conformation traits appear in both indices, but with a slightly modified trait complex definition (RZEn vs. RZEo), and three new trait complexes, mainly providing a better representation of health and welfare related traits, have been added in the new index. The former trait complex RZS, representing somatic cell score as an auxiliary trait for udder health, is now included directly in the trait complex ‘Health’ (RZH). The change of index weights reflects the increased emphasis on health and welfare in the breeding goal: while in the old index, milk yield (RZM) had a weight of 45%, it now has a weight of 36% and much of this weight has been relocated to welfare related trait complexes, especially by assigning 18% of the total weight to the new health trait complex (RZH).

We first apply the suggested methodology in detail to the old index, and later analyze the expected effects of the change from the old to the new index.

**Fig. 1:**
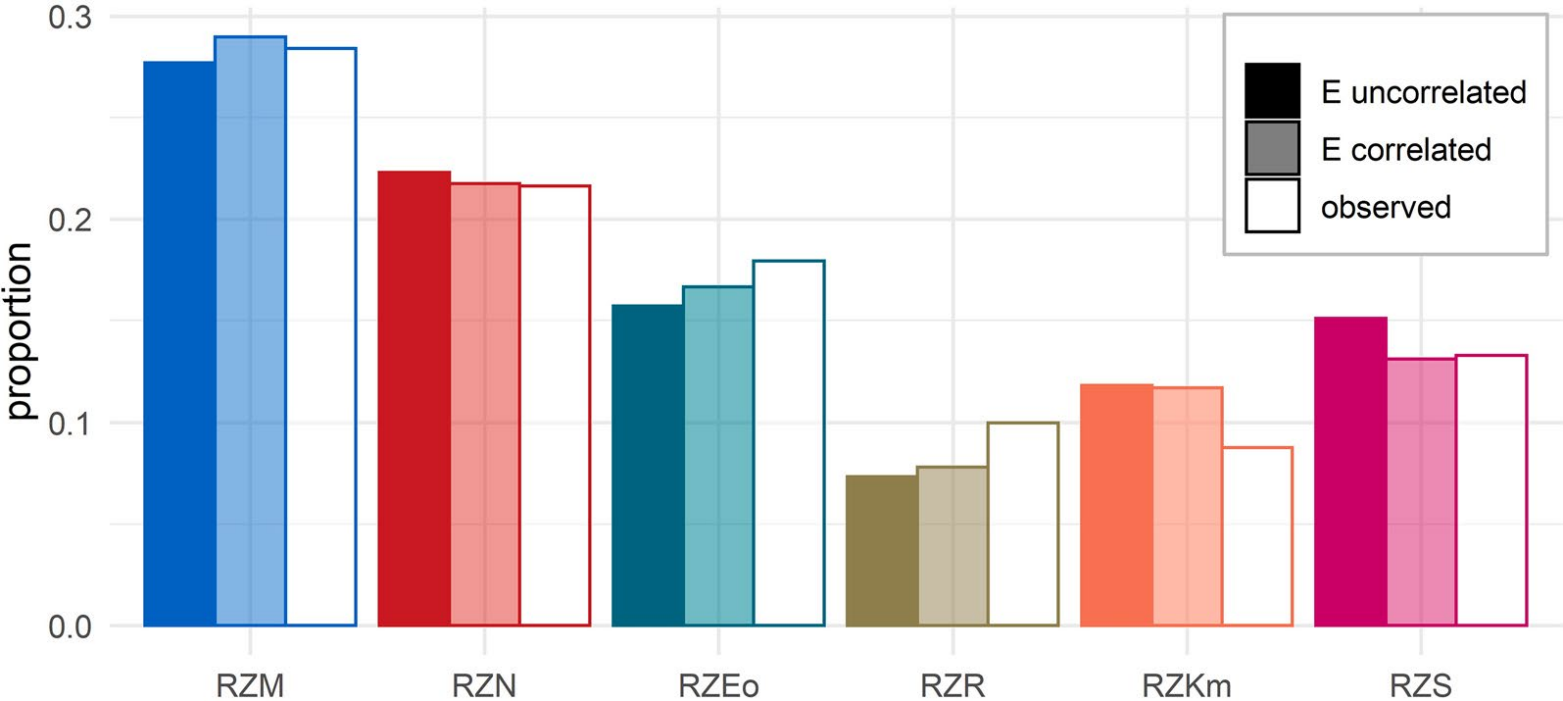
Expected composition of genetic trend without (E uncorrelated) and with (E correlated) accounting for the covariance between residuals, and observed composition of genetic trend for the six index trait complexes. Within one category, all proportions sum up to 1. For abbreviations of trait complexes see Table 1

The old index with six trait complexes was continuously used since August 2010, when genomic breeding value estimation was officially implemented for the Holstein breed in Germany, with only minor modifications in trait complex definitions and technical details of the trait-specific evaluation procedures since then. For these six trait complexes, we calculated observed genetic trends by regressing the average breeding values of bulls used in a certain year on the average birth year of those bulls, interpreting the slope of this regression as the genetic trend. This was done with data from 2010 to 2020, comprising bulls of the average birth years 2003 to 2016. The composition of the observed trend is displayed in the right column of Fig. 1.

### Estimation of the variance–covariance matrix of residuals

We estimated the variance–covariance matrix of the residual errors $\widehat{{\varvec{\Sigma}}}$ for the old index using the empirical covariance of sets of genomic breeding values of 108,458 German Holstein cows born in 2018. We preferred to use the covariance of breeding values of cows rather than that of bulls since breeding values for cows are much more numerous and arguably are less subject to bias caused by pre-selection. The correlation structure for the estimated genomic breeding values of cows is in the upper triangle of Table 2. From this, we calculated the residual variance–covariance matrix $\widehat{{\varvec{\Sigma}}}$ using Eq. (2), the residual correlations corresponding to this covariance matrix are in the lower triangle of Table 2. Note that 7 out of 15 correlations of the residual errors are negative and some are distinctly different from 0 (e.g. 0.47 or 0.36 for the correlation of residual errors between RZN and RZS or RZN and RZR, respectively).

**Table 2:** Empirical correlations between estimated breeding values (above the diagonal) and estimated correlations between residuals of estimated breeding values (below the diagonal)

	RZM	RZN	RZEo	RZR	RZKm	RZS
RZM		0.06	0.06	−.20	0.05	0.03
RZN	−.11		0.14	0.40	0.20	0.46
RZEo	−.10	−.03		−.03	0.03	0.15
RZR	−.31	0.36	−.10		0.30	0.13
RZKm	−.08	0.11	− .08	0.27		0.11
RZS	0.00	0.47	0.06	0.10	0.07	

For trait complex abbreviations see Table 1

### Composition of expected vs. observed genetic trend

We used Eq. (5) to calculate the expected composition of genetic progress with or without accounting for the covariance of residual errors. The results are displayed in Fig. 1. Overall, the expected and observed composition of genetic progress appear to match reasonably well, the largest discrepancies being observed for the functional trait complexes fertility (RZR) and maternal calving traits (RZKm). We observe that there are a number of differences between the two approaches used to derive the expected composition, with better overall agreement between expected and observed values when the covariance of residual errors is taken into account. Therefore, and because it is conceptually more appealing, we focus below on the results obtained with this approach. The largest discrepancies between expected and observed values are seen for the conformation and fertility traits, both of which show more genetic progress than expected, while genetic progress in calving traits lags behind expectations.

There are several possible reasons that explain these differences between the expected and the observed composition of the genetic trend:

- The main assumption underlying the calculation of the expected genetic progress is that all selection decisions are made based on the combined index. However, in real life, other criteria can be used: for each bull, not only the estimated breeding value for the combined index is published, but also estimated breeding values for each trait category as well as average daughter performances. Hence, breeders may decide to use a certain bull based on such detailed information, e.g. they may strive to focus on a specific trait complex, or they may prefer bulls from a certain line or origin, regardless of their actual breeding values.
- In mating software, breeders can configure their own overall index, whereby the official total merit index is preset. Internal evaluations of breeders' individual total indices in such mating software have shown that breeders give on average a greater weight to conformation traits (RZE) in their own index than in the official total merit index RZG.
- Furthermore, not all genetic progress comes from the selection of young bulls. In modern dairy cattle breeding programs, a small proportion of the overall genetic progress comes from the selection of cows [23, 24]. This proportion tends to increase as the number of genotyped cows increases. Especially the within-herd replacement of cows is often based on ad-hoc criteria—possibly linked to fitness-related traits—rather than on elaborated genomic breeding values. In the dataset used, there is also a certain proportion of progeny-tested bulls with significantly higher reliability. However, their impact decreased over time.
- We can even assume some effect of natural selection in fitness-related traits [25], especially on the cow selection paths, since, e.g. cows with poor fertility or inferior longevity will contribute less to the next generation.
- Finally, the expected values are derived under the assumption that the assumed parameters are correct; if this is not the case, the predictions may be biased [26]. This concerns especially the assumed correlations and reliabilities of the breeding values, which are estimated statistically and may therefore be subject to estimation errors.

However, it is very clear that there is no immediate link between the predefined weights in $\mathbf{w}$ and the genetic trend in $\mathbf{d}$. While e.g. milk was assigned 45% of the weight in the breeding goal until 2021, only about 28% of both, the expected and observed genetic trend pertain to this trait complex. On the other side of the spectrum, maternal calving traits are assigned just 3% of the total economic weight, but account for 8 or 12% of the observed and expected genetic trend, respectively. These discrepancies are inevitably caused by the construction of the index from breeding values of varying reliability. Nevertheless, in practice, it is often mistakenly assumed that the weights of vector $\mathbf{w}$ reflect the composition of the expected genetic progress in the trait complexes.

### Sensitivity of results to changes in economic weights

When designing an index, it may be of interest to see what effect it has on the composition of the genetic trend if a particular trait complex is weighted more or less. Obviously, we would expect a greater share of the genetic trend in a given trait complex if we assign more weight to it. However, due to the covariance structure and to the fact that assigning more weight to one trait reduces the relative weight of the other traits, we also expect a correlated response in other trait complexes. Tables 3 and 4 show these changes for the old and the new index, respectively, obtained as the approximated first derivative of the genetic trend with respect to the vector of economic weights calculated as described in the Methods section, using a marginal change by $\theta =0.001$. Results are reported in a standardized form, such that the absolute values in each line sum up to 1.

**Table 3:** Approximate change of genetic trend in all considered trait complexes when the relative economic weight of a single trait complex is marginally increased, as obtained by the approach described in the methods section with ${\varvec{\theta}}=0.001$ for the old index

Change in ${\mathbf{w}}_{\mathbf{u}}$ for trait complex	Relative expected change of genetic trend in trait complex					
	RZM	RZN	RZEo	RZR	RZKm	RZS
RZM	*0.21*	−.22	−.14	−.18	−.08	−.17
RZN	−.23	*0.38*	−.01	0.18	0.04	0.16
RZEo	−.21	−.01	*0.67*	−.05	−.03	0.04
RZR	− .18	0.16	− .03	*0.45*	0.13	0.04
RZKm	−.10	0.05	−.02	0.17	*0.64*	0.01
RZS	−.18	0.14	0.02	0.04	0.01	*0.60*

Results are scaled such that all absolute values in each line sum up to one. Diagonal values in italics represent the direct change in the modified trait complex, all other values are indirect changes in other trait complexes. For trait complex abbreviations see Table 1

**Table 4:** Approximate change of genetic trend in all considered trait complexes when the relative economic weight of a single trait complex is marginally increased, as obtained by the approach described in the methods section with ${\varvec{\theta}}=0.001$ for the new index

Change in ${\mathbf{w}}_{\mathbf{u}}$ for trait complex	Relative expected change of genetic trend in trait complex							
	RZM	RZN	RZEn	RZR	RZKm	RZKd	RZH	RZC
RZM	*0.26*	−.17	−.11	−.17	−.04	−.04	−.18	−.03
RZN	−.24	*0.24*	−.03	0.17	0.03	0.05	0.19	0.05
RZEn	−.16	−.03	*0.50*	−.05	−.05	−.10	−.01	−.10
RZR	−.18	0.12	−.03	*0.34*	0.12	0.07	0.12	0.02
RZKm	−.07	0.03	−.05	0.19	*0.56*	−.05	0.03	0.02
RZKd	−.06	0.05	−.11	0.10	−.05	*0.54*	0.06	0.03
RZH	−.26	0.19	−.01	0.16	0.03	0.06	*0.27*	0.04
RZC	−.07	0.07	−.13	0.03	0.02	0.04	0.05	*0.59*

Results are scaled such that all absolute values in each line sum up to one. Diagonal values in italics represent the direct change in the modified trait complex, all other values are indirect changes in other trait complexes. Note that residual correlations for the new index were assumed to be zero, as they could not be estimated from the data. For trait complex abbreviations see Table 1

Interestingly, on average across all trait complexes, just about half (49.1% for the old index, Table 3) or even less than half (41.2% for the new index, Table 4) of the expected changes in genetic trend are expected in the trait complex for which the economic weight is modified, ranging from 21% for milk to 67% for conformation trait complexes (both in Table 3). Furthermore, increasing the relative weights of the two trait complexes, milk and confirmation, is found to have a negative effect on the genetic trends of nearly all the other trait complexes, while increasing the weights of all other trait complexes has a positive effect on the genetic trend of most other trait complexes, except milk and conformation. The results also reflect nicely the ‘composite’ structure of the longevity trait complex: a greater economic weight for this trait complex affects RZN directly to 38 (24)% in the old (new) index, but has substantial positive effects on other trait complexes, such as fertility and udder health, which are known to be major causal factors for longevity [27].

As expected, the overall expected genetic progress with the marginally changed weight vector was found to be reduced (results not shown). Note that changes in the economic composition of an index can have a negative effect on the overall efficiency of a breeding program, especially, if important trait complexes are omitted or unimportant trait complexes are given importance, or when the direction of selection is reversed for an important trait complex [28].

### Expected composition of the phenotypic trend

**Fig. 2:**
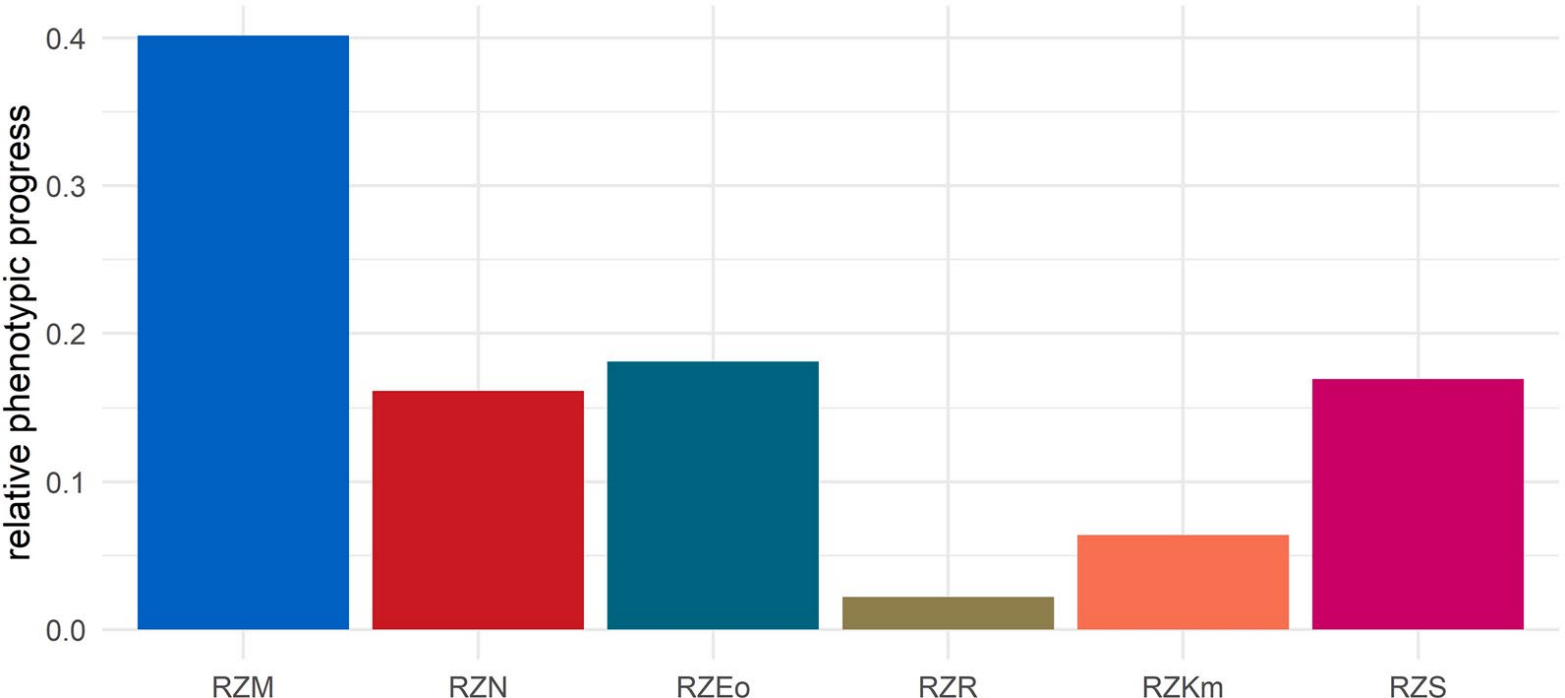
Expected composition of the phenotypic progress through selection in the six considered trait complexes. The values sum up to 1. For abbreviations of trait complexes see Table 1

The composition of the genetic trend cannot be directly converted into the composition of the phenotypic trend, because genetic progress by one genetic standard deviation has a smaller effect on the phenotypic trend for a trait complex with a low heritability than for a trait complex with a high heritability. The composition of the expected phenotypic trend, scaled in phenotypic standard deviations and resulting from the application of Eq. (6) is displayed in Fig. 2. Here, we see that 40% of the expected phenotypic trend caused by selection is accounted for by the trait complex 'milk', while the phenotypic trends in the trait complexes longevity (16.1%) and, in particular, fertility (2.2%) lag behind the composition of the expected genetic trend. These results illustrate that for trait complexes with a low heritability, the leverage of selection is very limited because most of the observed phenotypic variance is not genetically determined and thus cannot be influenced by breeding. This underlines the role of other fields of action such as management, feeding and hygiene when it comes to improving lowly heritable traits. However, even if the amount of phenotypic progress per year or generation through breeding is small, it must be remembered that genetic progress is cumulative and therefore sustainable phenotypic progress can be achieved over time.

### Realized economic weights

**Fig. 3:**
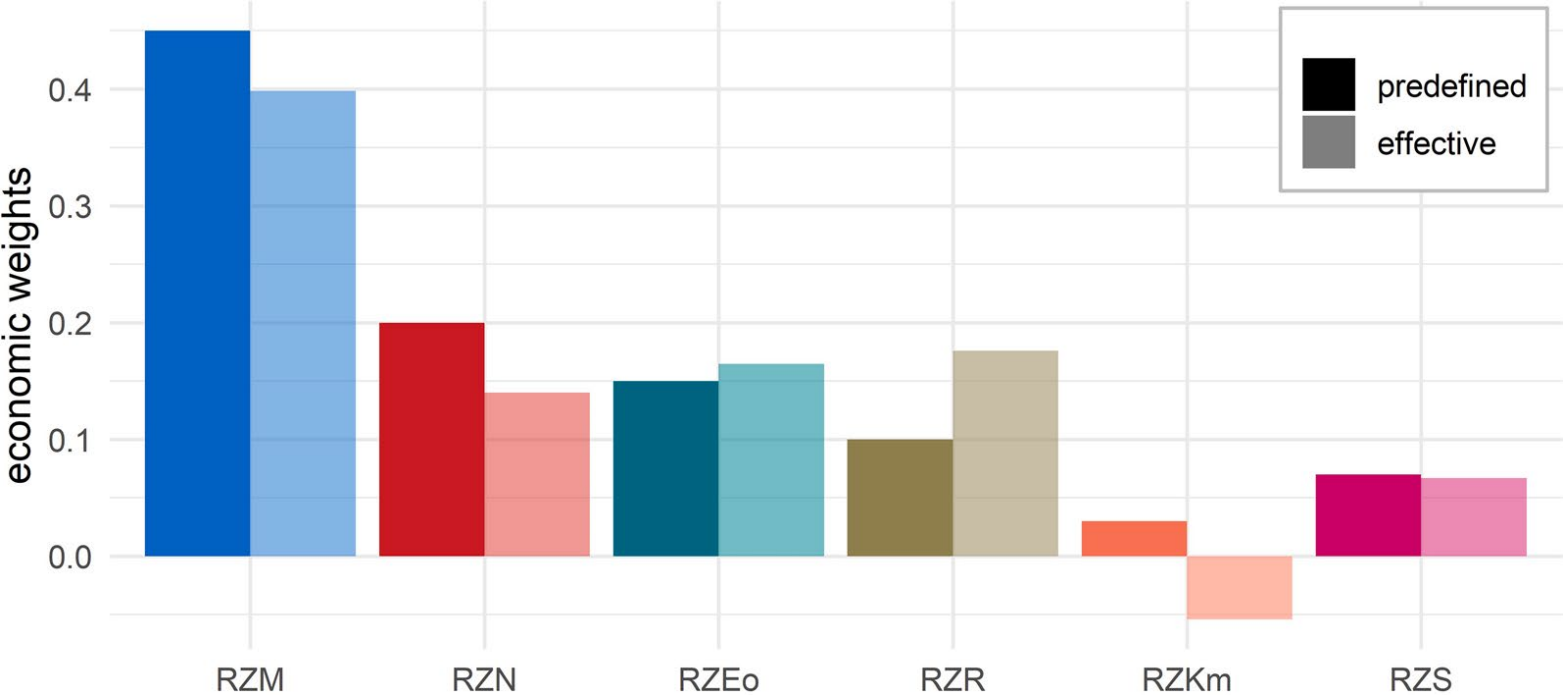
Predefined vs. effective economic weights for the six considered trait complexes. Within one category, all absolute values sum up to 1. For abbreviations of trait complexes see Table 1

The empirically observed genetic trend in the six traits of the index applied over the last 10 years can be used to derive the realized economic weights ${\varvec{\upomega}}$ [Eq. (8)], which are the weights that correspond best with the observed composition of the genetic trend. These effective economic weights are contrasted with the predefined economic weights in Fig. 3. On the one hand, not surprisingly, realized weights are greater than the predefined weights for trait complexes for which the observed trend is clearly exceeding the expected one, and especially for RZE and RZR ; for the latter the realized weight is 75% more than the predefined one. On the other hand, the realized weight for longevity is only 70% of the predefined one, and that for maternal calving ability is even negative. These results suggest that the relative value that is implicitly assigned to some of the trait complexes by breeders through their actual selection decisions deviates considerably from the predefined values, which are based on an economic analysis of the production system in combination with strategic decisions of the breeding associations.

### Expected effects of the change from the old to the new index

Finally, the suggested methodology was applied to assess the impact of modifications in the construction of an index. Starting in April 2021, a new total merit index was introduced, which comprises eight trait complexes (see Table 1). We used the suggested framework to compare the expected genetic trend in the new set of trait complexes when we either apply the old or the new index, basically revealing the impact of the change to the new index. Since we do not have comprehensive sets of empirical breeding values for all new trait complexes, and thus cannot estimate the covariance matrix of the estimation errors applying Eq. (2), this is done based on the assumption that residual errors of estimated breeding values are uncorrelated.

**Fig. 4:**
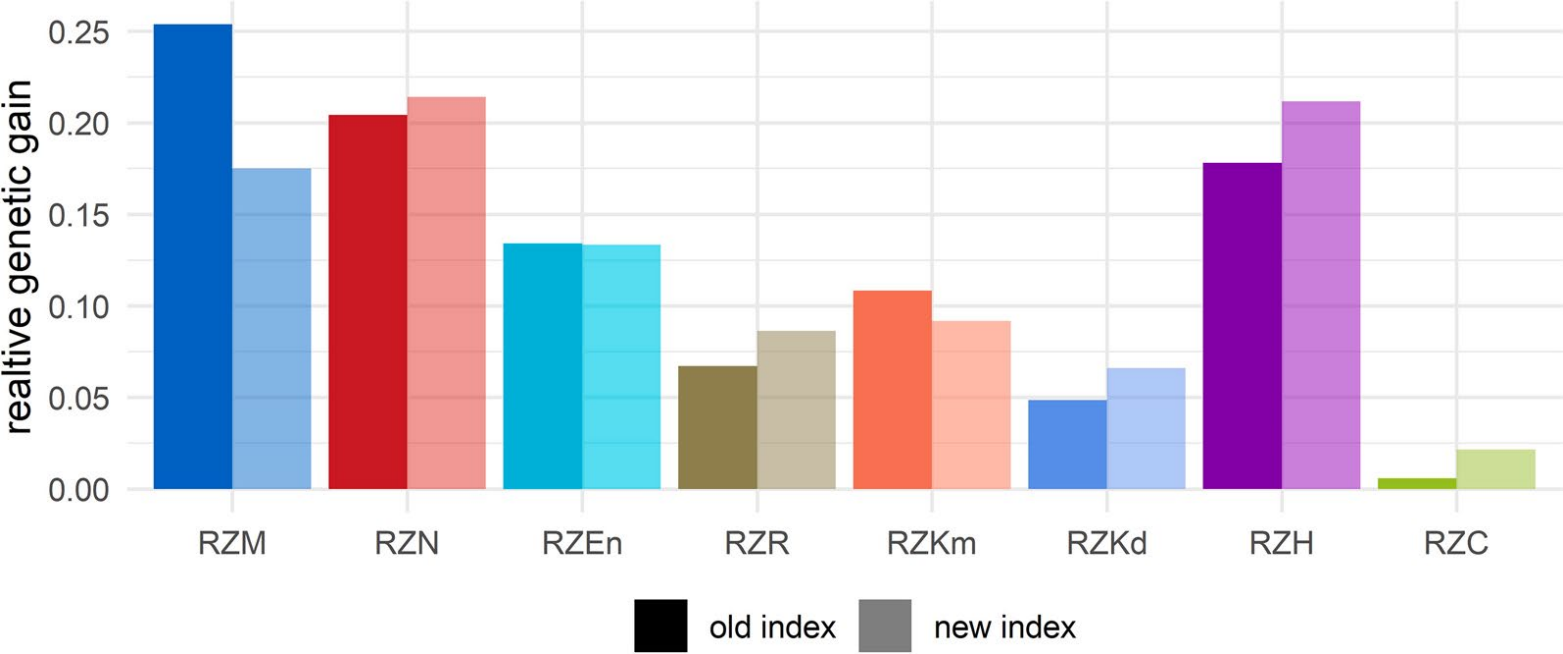
Expected composition of the genetic trend for the eight traits of the new index, when selection was done based on the old or the new index, respectively. Within one category, all values sum up to 1. For abbreviations of trait complexes see Table 1

Figure 4 contrasts the composition of expected genetic trend in the eight new traits when either the old or the new index is applied for selection. With the old index (the solid bars), we predict exactly the same genetic trend in absolute terms for the four traits that are represented in both indices, however, their proportion in the composition of the total genetic trend is reduced because the new index is made up by more - eight instead of six - traits than the old one. For the new index traits that are not present in the old index, we predict a correlated selection response.

Changes when switching to the new index can be attributed to several reasons: all eight traits are considered directly as index traits in the new index, different economic weights are applied, and more information on fitness-related traits is directly used for selection. As a consequence, productivity (RZM) loses importance (from 25.4 to 17.5% of expected genetic gain), which presumably is mainly due to the lower economic weight. The new fitness-related traits, RZH, RZKd and RZC, gain importance in the new index since they are directly observed and have been assigned an economic weight. Expected genetic gain in both RZN and RZR increase in spite of the fact that their relative economic weight has been reduced compared to the old index. Presumably, this is due to the inclusion of the health trait RZH, which shows a strong positive genetic correlation both with RZN (+ 0.78) and RZR (+ 0.41). As shown in Table 4, a change in weight of RZH has substantial effects on genetic trend in other traits, especially for RZN: when increasing the weight for RZH, the indirect effect (+ 0.19) on the genetic trend in longevity (RZN) is almost as high as the direct effect (+ 0.27) in RZH (actually, this phenomenon can be observed in both directions).

## Conclusions

We present a general framework for analyzing the effects of setting an overall breeding objective in genomic selection in complex breeding programs. The proposed approach is based entirely on classical selection index theory [4], but adopts some more recent concepts, such as combining estimated breeding values for some trait complexes into an overall breeding value, which requires consideration of the precision and covariance structure of lower-level estimates. We present a new approach to estimate the covariance structure of estimation errors from the empirical covariance structure of estimated breeding values and to derive realized economic weights from observed genetic trends. It should be noted that the determination of weights for complex breeding objectives cannot be considered a purely economic exercise, but in practice also takes into account whether the resulting genetic trends are acceptable to stakeholders, i.e., farmers, breeding associations, consumers or society. Therefore, the proposed framework and the analytical tools provided and implemented in the R package “IndexWizard”, may be helpful to define more rational and generally accepted breeding objectives in the future.

## Data Availability

The datasets supporting the conclusions of this article are included within the article and its additional files. The analysis script to derive all results using the R package “IndexWizard” can be found on GitHub (https://github.com/johannesgeibel/IndexWizard).
